# Properdin: a tightly regulated critical inflammatory modulator

**DOI:** 10.1111/imr.12466

**Published:** 2016-10-26

**Authors:** Adam Z. Blatt, Sabina Pathan, Viviana P. Ferreira

**Affiliations:** ^1^ Department of Medical Microbiology and ImmunologyUniversity of Toledo College of Medicine and Life SciencesToledoOHUSA

**Keywords:** complement, factor H, inflammation, properdin

## Abstract

The complement alternative pathway is a powerful arm of the innate immune system that enhances diverse inflammatory responses in the human host. Key to the effects of the alternative pathway is properdin, a serum glycoprotein that can both initiate and positively regulate alternative pathway activity. Properdin is produced by many different leukocyte subsets and circulates as cyclic oligomers of monomeric subunits. While the formation of non‐physiological aggregates in purified properdin preparations and the presence of potential properdin inhibitors in serum have complicated studies of its function, properdin has, regardless, emerged as a key player in various inflammatory disease models. Here, we review basic properdin biology, emphasizing the major hurdles that have complicated the interpretation of results from properdin‐centered studies. In addition, we elaborate on an emerging role for properdin in thromboinflammation and discuss the potential utility of properdin inhibitors as long‐term therapeutic options to treat diseases marked by increased formation of platelet/granulocyte aggregates. Finally, we describe the interplay between properdin and the alternative pathway negative regulator, Factor H, and how aiming to understand these interactions can provide scientists with the most effective ways to manipulate alternative pathway activation in complex systems.

This article is part of a series of reviews covering Preformed Mediators of Defense appearing in Volume 274 of *Immunological Reviews*.

## 1 Introduction

While the basic principles of complement activation are well‐characterized, complex interactions between complement and other cells and proteins, especially in the context of disease pathogenesis, have precluded, with few exceptions, successful use of anti‐complement therapeutics in the clinic for common diseases. Highlighting a difficulty in understanding the complete scope of complement activity is the intricate interplay between regulatory mechanisms that govern complement activation. While most human cells and tissues express membrane‐bound regulatory proteins to protect themselves from complement attack, soluble regulatory proteins are recruited to inflammatory sites through interactions with both complement proteins and other ligands defined by the unique composition of the local microenvironment. The soluble complement protein properdin is both a regulator and an initiator of the complement alternative pathway that has potent effects on the level of complement activation. Understanding the complex biology of properdin, however, has proven difficult due to the different sources of properdin used in biochemical studies and its intricate self‐associations. Despite these difficulties, experimental data have proven properdin to be both a powerful inflammatory modulator that can greatly enhance tissue‐damaging inflammation and a critical promoter of microbial clearance in multiple disease models. Here, we discuss basic principles of, and recent advances in understanding, properdin biology, highlighting areas that require future studies. We progress to describe the interplay between properdin and the negative regulator Factor H, the potential of properdin inhibitors in treating human disease with an emphasis on the role of properdin in thromboinflammation, and mention potential consequences of properdin inhibition as evidenced in animal models. Finally, we briefly elaborate on the utility of using novel strategies to promote properdin activity on the surface of microorganisms. To facilitate discussion, we begin with a condensed review of the complement system, which has been extensively reviewed elsewhere.^1^, ^2^, ^3^

## 2 The complement system

The complement system is a group of soluble blood proteins that rapidly activate in a cascade‐like manner to orchestrate inflammatory and immune responses in the human host. Complement activity can be initiated by one of three pathways termed the classical, lectin, and alternative pathways, which all converge at the cleavage of the central protein C3 by an enzymatic complex called the C3 convertase. C3 cleavage releases the small fragment C3a, and the resulting larger fragment, C3b, can bind covalently to nearby hydroxyl‐ and amino‐groups via an exposed thioester bond. C3b bound to, or in close proximity to, the C3 convertase changes the specificity of the complex, allowing for the cleavage of C5 to C5a and C5b. The terminal pathway activates through a series of conformational changes in complement proteins, initiated by the binding of C5b to C6, followed by sequential binding of C7, C8, and C9 that results in the formation of the membrane attack complex (MAC; C5b‐9).^1^, ^3^

The classical and lectin pathways utilize distinct initiation mechanisms that lead to the generation of the same C3 convertase, denoted C4b2b. Classical pathway activation is primarily a result of antibody‐antigen interactions that promote the activation of the C1 complex, while the lectin pathway activates in response to recognition of specific carbohydrate patterns by various lectin molecules. The alternative pathway, whose activation mechanism will be discussed below, is unique among the three complement pathways because it can activate without the requirement for specific antigen recognition.^2^ Each complement pathway generates the same set of effector molecules to carry out its functions. C3b and its breakdown products iC3b and C3dg tag surfaces for recognition by complement receptors expressed on human cells (CR1, CR2, CR3, CR4, and CRIg).^1^ The anaphylatoxins, C3a and C5a, bind to the G‐protein‐coupled receptors C3a receptor (C3aR) and C5a receptor 1 (C5aR1; CD88), respectively, to promote inflammation and other diverse functions, including cerebellar development, homing of stem cells to the bone marrow, and tissue fibrosis.^4^ The MAC is capable of directly forming pores in membranes, leading to direct microbial killing, and sublytic levels of MAC stimulate a variety of proinflammatory responses in human cells, including cell and inflammasome activation.^5^

### 2.1 The alternative pathway

**Figure 1:**
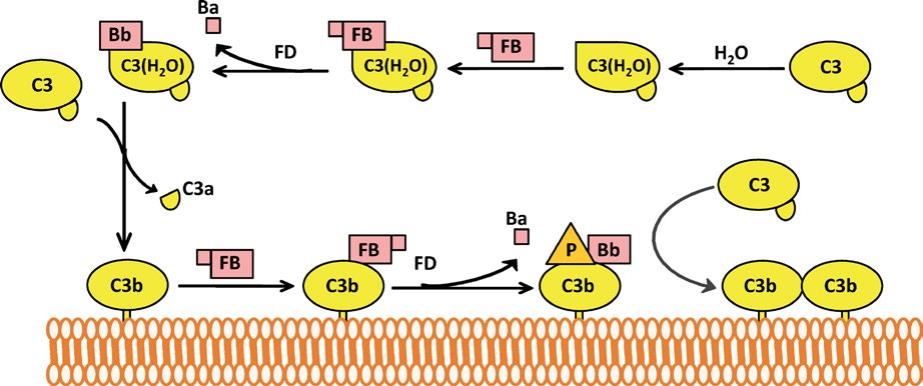
Activation of the alternative pathway. *Described from upper right to left:* The thioester bond in C3 is spontaneously hydrolyzed by water, leading to the formation of C3(H_2_O), which can recruit Factor B (FB). Once bound to C3(H_2_O), FB is cleaved by Factor D (FD) to Bb to form the alternative pathway fluid phase C3 convertase, C3(H_2_O)Bb. The C3 convertase cleaves C3 to C3a and C3b, which can bind covalently to nearby amino‐ and hydroxyl‐groups via its thioester group. C3b covalently bound to a surface recruits FB, which is subsequently cleaved by FD to form the alternative pathway cell‐surface C3 convertase, C3bBb. While C3bBb has a half‐life of only approximately 90 seconds, properdin (P) stabilizes the convertase to increase its activity 5‐ to 10‐fold

**Figure 2:**
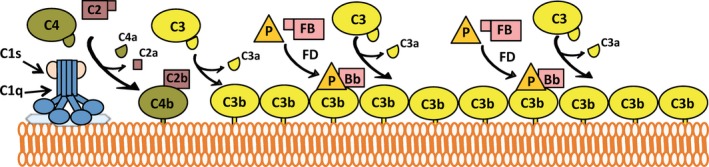
Alternative pathway amplifies all complement activity. C3b originally deposited on a surface by the classical pathway can act as a site for formation of the alternative pathway cell‐surface C3 convertase. The alternative pathway deposits more C3b on the surface, which can act as additional sites for alternative pathway C3 convertase formation. Therefore, even minor complement activity initiated by the classical (or lectin) pathway can be quickly and efficiently amplified by the alternative pathway

The alternative pathway continuously activates at a low level in the fluid phase of blood due to spontaneous hydrolysis of the labile thioester bond in C3, in a process known as ‘tick‐over’. An additional source of C3(H_2_O) may be formed by contact activation on certain cells, such as platelets and artificial surfaces.^6^, ^7^ Upon hydrolysis of its thioester bond, C3 undergoes a conformational change to form C3(H_2_O) and gains the ability to bind Factor B.^8^, ^9^, ^10^ Factor B in association with C3(H_2_O) is cleaved by Factor D to Ba and Bb, to generate the fluid‐phase alternative pathway C3 convertase, C3(H_2_O)Bb. C3(H_2_O)Bb cleaves additional C3 molecules to C3a and C3b, exposing the labile thioester bond on C3b.^8^ The thioester bond is rapidly hydrolyzed by water, and only a small percentage of C3b is able to bind covalently to nearby hydroxyl‐ and amino‐groups on surfaces before hydrolysis.^11^ Covalently bound C3b tags surfaces for additional alternative pathway activation. Surface‐bound C3b, which is structurally similar to C3(H_2_O), recruits Factor B molecules, which are cleaved by Factor D in an identical manner to that which occurs in the fluid phase.^12^ The surface‐bound alternative pathway C3 convertase, C3bBb, cleaves more C3 molecules to C3a and C3b, which, if bound covalently to the surface, act as focal points for the assembly of more alternative pathway convertases, effectively amplifying its own activity.^13^ Figure 1 summarizes alternative pathway activation. C3b initially deposited by the classical or lectin pathways can also serve as sites for alternative pathway activation; therefore, the alternative pathway is an amplification loop for all complement activity. The alternative pathway may account for approximately 80% of terminal pathway activity even when complement is initially activated by the classical 14 or lectin pathways 15 (Fig. 2).

### 2.2 Alternative pathway regulation

**Figure 3:**
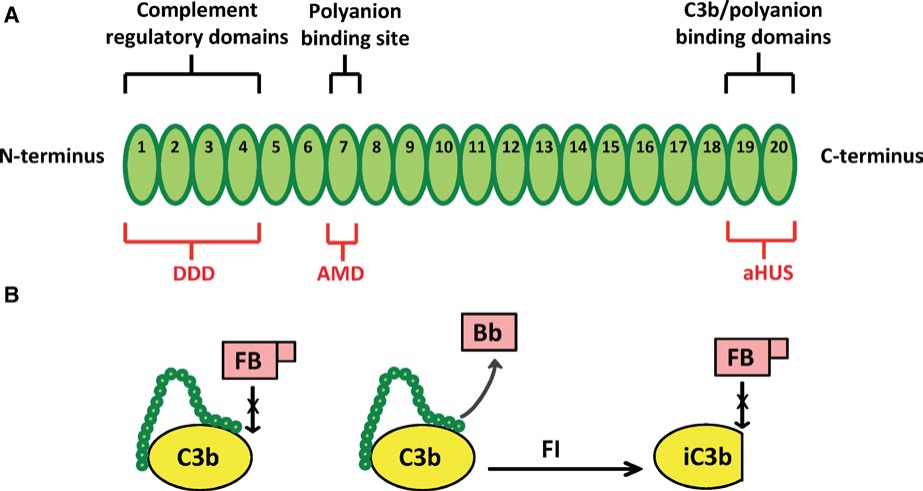
Factor H structure and function. (A) Simplified schematic representation of Factor H and disease associations. Factor H is composed of 20 homologous complement control protein (CCP) domains. The N‐terminal 4 domains bind C3b and contain the regulatory functions of Factor H, while the C‐terminal domains 19–20 bind both C3b and polyanions to anchor Factor H to cell surfaces. Dense deposit disease (DDD) occurs when the N‐terminal domains are impaired or absent (due to Factor H deficiency), whereas most mutations in Factor H associated with atypical hemolytic uremic syndrome (aHUS) are found in the C‐terminus, resulting in defective cell‐surface alternative pathway regulation. The Y402H polymorphism in domain 7 is strongly associated with the development of age‐related macular degeneration (AMD). (B) Factor H regulatory functions. Factor H N‐terminal domains 1–4 regulate the alternative pathway via three different mechanisms: (left) competing with Factor B (FB) for binding to C3b; (middle) accelerating the decay of the alternative pathway C3 convertase; and (right) acting as a cofactor for FI‐mediated cleavage of C3b to iC3b, a C3 fragment that cannot bind FB

Its spontaneous nature and its ability to amplify all complement activity make regulation of the alternative pathway a necessity in the human host in order to prevent excessive inflammation and tissue damage. Human cells and tissues are protected from complement attack by various membrane‐bound complement regulatory proteins, including CD55, CD59, CD46, and CR1.^1^ However, the serum glycoprotein, Factor H (Fig. 3A), which has been reviewed elsewhere,^16^ has also proven to be critical to limiting alternative pathway activation on the surface of several cell types, even in the presence of membrane‐bound regulators. Factor H is also the primary regulator of the alternative pathway in the fluid phase, preventing complement consumption via uncontrolled alternative pathway activation.

**Figure 4:**
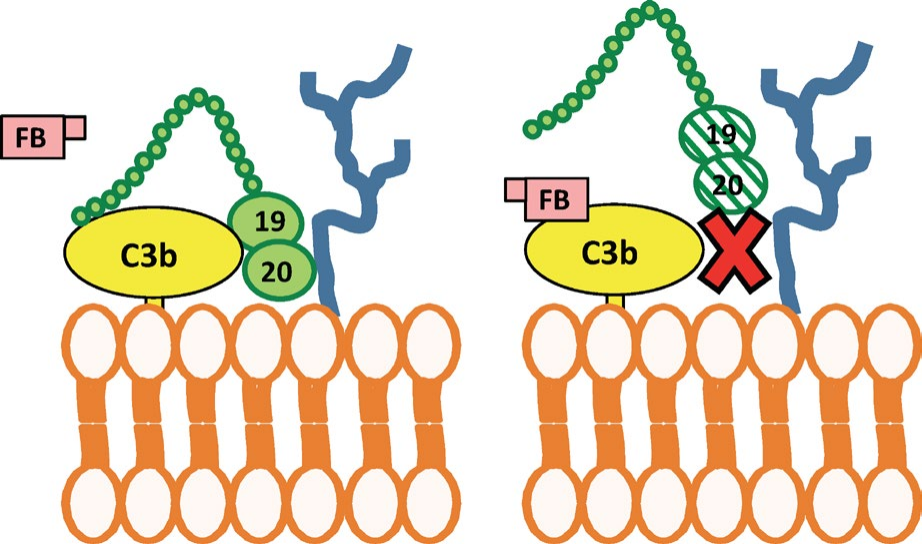
C‐terminal domains 19–20 mediate Factor H cell‐surface protection. (Left) Factor H domains 19–20 bind simultaneously to C3b and polyanions (blue lines) on cell surfaces. These domains are the primary region of Factor H that anchors the protein to the cell surface to allow efficient alternative pathway regulation by N‐terminal domains 1–4. (Right) AHUS‐related mutations impair the ability of the domains 19–20 to bind to the C3b/polyanion combination on cell surfaces, and thus Factor H is dysfunctional in its ability to regulate the alternative pathway on cell surfaces, but not in the fluid phase

Factor H functions by accelerating the decay of the convertases by promoting the dissociation of Bb from C3(H_2_O) and from C3b,^17^, ^18^ as well as by acting as a cofactor for Factor I‐mediated cleavage of C3b and C3(H_2_O) to iC3b and iC3(H_2_O), respectively.^19^ The C3 fragments iC3b and iC3(H2O) cannot bind Factor B and can thus not form additional alternative pathway C3 convertases (Fig. 3B). Factor H consists of 20 domains and circulates in the blood at approximately 150–550 μg/mL.^20^ The regulatory functions of Factor H are contained within its four most N‐terminal domains,^21^, ^22^, ^23^ while its most C‐terminal domains, 19 and 20, are key for anchoring Factor H to cell surfaces.^24^, ^25^, ^26^, ^27^, ^28^, ^29^, ^30^, ^31^, ^32^, ^33^, ^34^ Dysfunction of the Factor H N‐terminal domains or low levels of circulating Factor H are associated with type II membranoproliferative glomerulonephritis, also known as dense deposit disease (DDD), characterized by insufficient fluid‐phase regulation that causes consumption of C3.^35^, ^36^ Contrarily, mutations primarily located in domains 19‐20 that limit Factor H‐mediated cell‐surface protection, but retain fluid‐phase regulation, are associated with the prothrombotic inflammatory disease atypical hemolytic uremic syndrome (aHUS)^30^, ^37^, ^38^, ^39^, ^40^, ^41^, ^42^, ^43^, ^44^ (Figs 3A and 4).

A recombinant protein composed of the two most C‐terminal domains of Factor H known as rH19‐20 can compete with Factor H on the cell surface, without impairing the ability of Factor H to regulate the alternative pathway in the fluid phase.^24^ By competing on the cell surface with endogenous Factor H present in whole blood, plasma, and serum, without affecting Factor H fluid‐phase regulation, rH19‐20 can be used in vitro and in vivo as an efficient tool to (i) evaluate the contribution of Factor H cell‐surface protection in inflammatory disease models 31, 45, 46, 47, 48 and (ii) enhance the understanding of the pathogenic mechanisms that lead to aHUS.^24^, ^38^, ^49^, ^50^, ^51^

Alternative pathway convertases are inherently unstable, with a half‐life of only approximately 90 seconds.^52^ Properdin, the focus of this review, has a well‐defined role in which it acts as a positive regulator of complement activity by stabilizing the alternative pathway convertase, thus increasing its activity 5‐ to 10‐fold.^53^ Properdin stabilizes the convertase by primarily binding C3b, although it makes additional contacts with Factor B and Bb.^54^, ^55^, ^56^ Properdin binds to C3bB and C3bBb with higher affinity than to C3b alone.^55^ Interestingly, properdin has been shown to directly limit Factor H cofactor activity^55^, ^57^ and is predicted to affect Factor H decay accelerating activities via structural data,^54^ suggesting that defective Factor H regulation may enhance the positive regulatory functions of properdin.

## 3 Molecular structure and characteristics of properdin

Properdin is a 53‐kDa monomer composed of six complete thrombospondin type 1 repeat (TSR) domains labeled TSR 1‐6^58^, ^59^ and a truncated N‐terminal TSR domain containing key conserved residues denoted TSR 0.^59^ Monomers, which are 26 nm in length × 2.5 nm in diameter,^60^, ^61^ consist of 442 amino acid residues, contain an N‐glycosylation site in TSR‐6,^62^ and are C‐mannosylated at 14 different tryptophans.^63^ Known binding ligands for properdin (other than C3b, C3bB, and C3bBb) include sulfatides,^64^ and various polyanionic structures discussed in a later section.

Under physiological conditions, properdin forms dimers (P_2_), trimers (P_3_), and tetramers (P_4_) in a defined 26:54:20 (P_2_:P_3_:P_4_) ratio via head‐to‐tail associations of its monomers.^65^ Mutant forms of properdin lacking single TSR domains revealed roles for the biological properties of individual domains.^62^ Domains 4, 5, and 6 are critical for the ability of properdin to mediate lysis of rabbit erythrocytes, which can be explained by completely abrogated binding to C3b and sulfatides by domain 5 and 6, or partially by domain 4, deletion mutants. Deletion of TSR 3 has no effect on binding to C3b or sulfatides nor oligomer formation. Domain 4 and 5 deletion mutants fail to form trimers and tetramers but retain the ability to dimerize, while deletion of TSR6 completely inhibits oligomerization.^62^ Collectively, the results from the study by Higgins et al.^62^ suggest that multiple TSR domains act in concert to mediate properdin oligomerization and function, with key roles implicated for domains 5 and 6. The critical nature of domains 5 and 6 for properdin functionality were supported by subsequent studies in which antibodies raised against human TSR5^66^ and mouse TSR5‐6 67 produced in *Escherichia coli* effectively inhibited properdin function in vitro and in vivo, respectively. Furthermore, the Y387D point mutation in TSR6 abrogated the ability of properdin to bind C3b and regulate the alternative pathway. This mutation is associated with type III properdin deficiency, characterized by severe functional impairment despite normal plasma levels of properdin.^68^ Two other mutations, R73W (TSR1) and Q316R (TSR5), are related to type II properdin deficiency, defined by very low levels of circulating properdin.^69^ Properdin recovered from these affected individuals had oligomerization defects and primarily consisted of dimers.^70^ Other previously identified mutations in the properdin gene primarily lead to type I properdin deficiency defined by a complete lack of circulating properdin,^69^ and thus these mutations have not provided useful insights into the domains/residues mediating properdin function.

**Figure 5:**
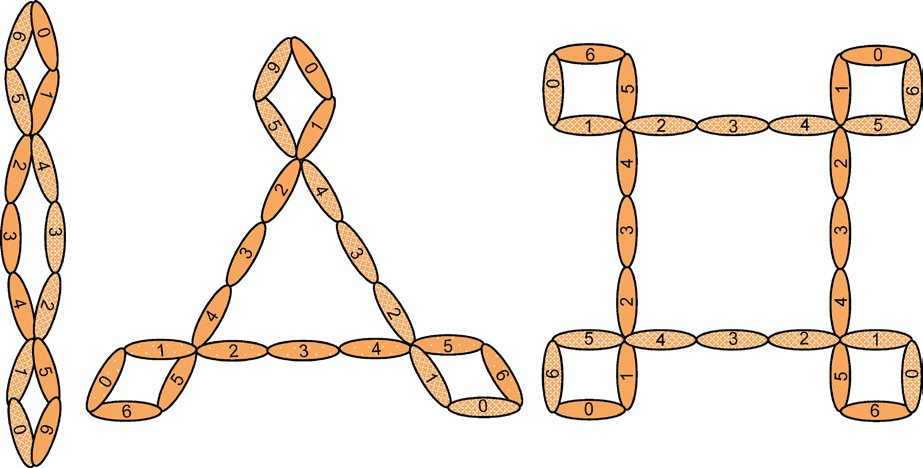
Properdin structure. Properdin monomers are composed of 7 thrombospondin repeat (TSR) domains labeled TSR0‐6. Under physiological conditions, properdin monomers form cyclic dimers, trimers, and tetramers in an approximately 1:2:1 ratio. Molecular modeling suggests that the vertices of properdin oligomers are composed of a total of four domains comprised from two different monomers in a head‐to‐tail organization. The exact organization of domains at the vertices has not been elucidated, but depicted here are theoretical models for oligomers in which the vertices are formed by TSR0‐1 from one monomer and TSR5‐6 from the other. Model proposed by Alorco et al^54^

Structural studies by Sun et al.^59^ and Alcorlo et al.^54^ proposed models for oligomerization. These studies illustrated roles for TSRs 0‐1 and 5‐6 in mediating contacts at the vertices of properdin oligomers, which is consistent with oligomerization defects described for domain 5 and 6 deletion mutants^62^ and for point mutations in domains 1 and 5.^70^ The exact composition of domains at the vertices could not be determined, with both studies citing a potential for variable combinations of domains.^54^, ^59^ Alcorlo et al.^54^ also proposed a ‘curly’ appearance of the vertices (Fig. 5), suggesting that unique epitopes could be found on different properdin oligomers due to the distinct geometrical constraints required for formation of individual oligomers. The structural models proposed in both studies also supported the roles for TSR5‐6 in mediating properdin function and were in agreement with the location and effect of the Y387D mutation on C3b binding.^54^, ^59^

## 4 Properdin sources

Properdin circulates in plasma at approximately 4–25 μg/mL and is unique among complement proteins in that it is primarily produced by leukocytes rather than hepatocytes.^71^ Many different leukocyte subsets synthesize properdin, including human T cells,^72^ monocytes,^73^ and mast cells.^74^ Mature neutrophils do not synthesize properdin but contain large stores of it in secondary granules, which are rapidly released upon stimulation with a variety of agonists, including C5a, fMLP, TNF‐α, and IL‐8.^75^, ^76^ A complete review of biological sources of properdin can be found in Cortes et al.^71^ Kimura et al.^77^ provided in vivo evidence for the role of myeloid cells in producing circulating properdin in a K/BxN model of murine arthritis; however, there are no studies suggesting which cell type primarily accounts for properdin in human plasma. In addition, very little is known about how different diseases affect the rate of clearance from the circulation. Ziegler et al.^78^ showed that higher levels of properdin were found in the urine of patients with renal disease vs healthy individuals, and Corvillo et al.^79^ proposed using serum properdin concentrations as a diagnostic marker for a subset of C3 glomerulopathy patients. Studies are lacking, however, that shed light on the dynamics of properdin metabolism in the context of diseases that do not affect the kidney or that have excessive leukocyte activation or leukocyte dysfunction.

Another key issue that remains unresolved is the molecular composition of properdin produced by different leukocyte sources. T‐cell‐derived properdin was shown to be approximately 100× more active than serum‐derived properdin in an alternative pathway hemolysis assay^72^; however, the reason for this enhanced functionality was never determined. Saggu et al.^31^ showed that physiological oligomers of properdin isolated from plasma or contained in the supernatants of PMA‐stimulated neutrophils could bind directly to platelets, but a direct comparison of the activity of equivalent amounts of neutrophil‐derived vs purified properdin was not conducted. Whether neutrophils secrete properdin oligomers in an approximate physiological ratio remains to be determined. Furthermore, the effect that pathophysiologic conditions have on the distribution of properdin oligomers remains unknown. Upon initial characterization, Pangburn^65^ demonstrated the ability of all properdin oligomers to redistribute to approximately the physiological ratio (26:54:20 P_2_:P_3_:P_4_) following denaturation‐renaturation cycles induced by guanidine or low pH. These data suggest that the ratio of properdin oligomers may remain relatively stable in solution even in the presence of inflammatory insults; however, the possibility remains that complex interactions with cells and other ligands in physiological or pathophysiologic states may change the ratio and/or activity of properdin.

## 5 Properdin as an initiator of alternative pathway activity: evidence and setbacks

**Figure 6:**
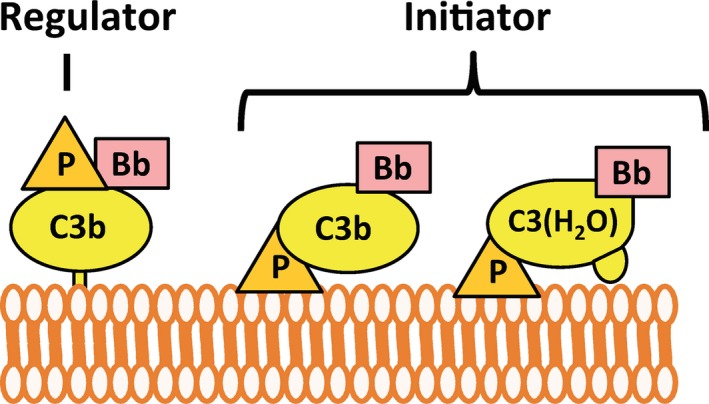
Properdin functions. Properdin (P) enhances alternative pathway activity in one of two ways: (1) acting as a positive regulator of pre‐existing alternative pathway activity or (2) initiating alternative pathway activity. Properdin acts as a positive regulator by stabilizing the alternative pathway C3 and C5 convertases, increasing their activity 5‐ to 10‐fold. As a pattern recognition molecule, properdin binds selectively to specific surfaces upon which it recruits C3b or C3(H_2_O) to initiate alternative pathway activity

When Pillemer first described properdin in 1954, he proposed it acted as an initiator of alternative pathway activity.^80^ This theory was later discounted and replaced with the well‐established role of properdin as a positive regulator of preexisting alternative pathway activity.^53^ Within the past decade, multiple studies have rediscovered the ability of properdin to act as an initiator of alternative pathway activity (31, 81, 82, 83, 84, 85, 86, 87, 88, 89, 90, 91 (Fig. 6). Caution must be taken in interpreting results from studies that utilized unfractionated, purified properdin (all studies referenced above except Vuagnut, Ferreira, Cortes, and Saggu et al.). Upon repeated freeze‐thaw cycles, purified properdin forms non‐physiological aggregates (P_n_) that have distinctly different properties from physiological oligomers. First, non‐physiological aggregates have ‘activated properdin activity’, meaning they induce alternative pathway activation in solution leading to complement consumption.^65^, ^92^ Second, non‐physiological aggregates bind non‐specifically to surfaces, including live Jurkat and Raji cells,^83^ *Neisseria* species,^93^ and non‐activated platelets.^31^ The physiological oligomers, however, can act as pattern recognition molecules binding selectively to specific surfaces, including zymosan, necrotic Jurkat and Raji cells,^83^ *Chlamydia pneumoniae,* 82 and activated platelets.^31^ The arrangement of properdin monomers in non‐physiological aggregates (described by Farries as ‘large amorphous aggregates’) remains unknown, but given the highly positively charged nature of properdin monomers, non‐specific ionic interactions with negatively charged anions could account for their robust binding abilities.

Unfractionated properdin isolated from plasma, which contains properdin polymers (P_2_, P_3_, P_4_, and non‐physiologicial aggregated P_n_) is visualized as approximately 53‐kDa monomers in SDS‐PAGE under both reduced and non‐reduced conditions. Although some studies detect purified properdin at high molecular weights (approximately 220 kDa) in SDS‐PAGE, and others have used SDS‐PAGE to rule out the presence of aggregates in their properdin preparations, this is inadequate because the polymeric forms (including the non‐physiological aggregates) are not visible on an SDS‐PAGE^83^ due to the non‐covalent nature of the polymer associations. The multimers can easily be detected, however, when the same unfractionated properdin preparations are separated by size exclusion or ion exchange chromatography.^83^ Interestingly, in a coimmunoprecipitation assay, Pauly et al.^94^ discovered C3(H_2_O), C3 fragments, clusterin, and immunoglobulins associated with properdin in human serum. Also, C3b_2_‐natural IgG complexes have been shown to stimulate complement amplification in a properdin‐dependent manner.^95^ These associations between properdin and other molecules may account for the properdin that migrates at a high molecular weight in SDS‐PAGE in some purified preparations. The nature of the interactions between properdin and these other molecules in human serum, which resist SDS, denaturing, and/or reducing agents, remains to be determined.

Differences have been noted in the binding capabilities of purified properdin vs properdin contained in serum. For instance, purified physiological properdin oligomers bound directly to zymosan in solution,^83^ but properdin required C3 activity to bind zymosan in the presence of serum in subsequent studies.^96^, ^97^ Similarly, binding of properdin trimers to thrombin‐activated platelets was inhibited in the presence of normal human serum.^31^ These results have led to the proposition that properdin binding is regulated in serum by unknown inhibitors. As mentioned above, C3(H_2_O), C3 fragments, clusterin, and immunoglobulins were associated with properdin in human serum.^94^ While the physiological effects of the interactions between these molecules and properdin remain unknown, it is possible that they could account for the qualitative differences in binding observed for purified properdin vs properdin contained in serum. Recent evidence also indicates that monomeric C‐reactive protein (mCRP) inhibits the binding of unfractionated properdin to proximal tubular epithelial cells.^88^ Because mCRP is generated and present mainly on cell surfaces or local microenvironments,^98^ it is likely that the inhibitory capacity of mCRP may be limited to cell surfaces and not affect properdin activity in normal human serum. The ability of serum to inhibit direct properdin binding can also be influenced by the specific ligands, as Xu et al.^91^ showed that unfractionated properdin and properdin in either normal human serum or C3‐depleted serum bound directly to necrotic cells to approximately the same extent. This adds another layer of complexity to properdin studies, indicating that direct properdin binding is influenced not only by the type of properdin used for the study but also by the specific ligands tested. Inhibitors in serum may fine‐tune properdin‐initiated functions by limiting direct properdin binding to some, but not all, surfaces.

Because of the ability of neutrophils to secrete properdin from their secondary granules upon stimulation,^76^ the concentration of properdin in inflammatory microenvironments is likely higher than the circulating concentration. This suggests that locally produced properdin could bypass regulatory mechanisms that exist in serum thus enabling properdin‐initiated alternative pathway activation. Indeed, Kemper et al.^86^ demonstrated that apoptotic T cells bound unfractionated and neutrophil‐derived properdin but not properdin from C3‐depleted serum. In addition, Saggu et al.^31^ demonstrated an ability of neutrophil‐derived properdin to bind directly to activated platelets. Conversely, Harboe et al.^97^ showed that neutrophil‐derived properdin was incapable of binding directly to solid‐phase zymosan, a surface previously shown to directly bind physiological properdin oligomers.^83^ The discrepancies between the latter two studies may be due to the different methodologies used to determine properdin binding (solid‐phase ELISA^97^ vs flow cytometry),^83^ or it could be an indication that there are indeed functional differences between physiological properdin oligomers and neutrophil‐derived properdin, as discussed in the former section.

A considerable obstacle for understanding properdin biology has been the inability to differentiate between initiating and regulating functions of properdin in vivo and ex vivo. Zhou et al.^99^ used an indirect approach in a murine model of abdominal aortic aneurysm (AAA) to differentiate between properdin as an initiator or regulator of alternative pathway activity. In their study, Zhou et al.^99^ complemented a properdin‐deficient mouse (protected from AAA development) by injection of either wildtype Factor B or Factor B containing the gain‐of‐function mutation D276G that produces a stable convertase without the need for properdin. Injection of D276G Factor B effectively reestablished the AAA phenotype, indicating that the role for properdin in this model is likely due solely to its ability to act as a stabilizer of the alternative pathway convertase. This strategy offers a novel approach to differentiating the functions of properdin in vivo, provided the experiments include properdin‐deficient animals; however, it could not be utilized for ex vivo studies using human whole blood or plasma.

Identification of the precise ligands for direct properdin binding could lead to the development of inhibitors that block properdin‐initiated functions without impairing its role as a convertase stabilizer in vivo or ex vivo. Physiological (‘native’) properdin binds to the sulfatide [Gal(3‐SO_4_βl‐lCer], while dextran sulfate (500 000 mw) and fucoidan inhibit this interaction.^64^ Findings by Holt et al. suggest properdin may bind directly to negatively charged polyanionic structures in the human host to initiate alternative pathway activity. Subsequent studies have identified interactions between properdin and heparin,^100^ chondroitin sulfate C^86^ and E,^86^, ^101^ and heparan sulfates.^86^, ^101^, ^102^ However, all of these studies utilized purified, unfractionated properdin to investigate binding. Holt et al. noted differences in the binding of physiological (‘native’) vs non‐physiological (‘activated’) properdin to sulfatides, as well as differences in the GAG preparations that inhibited these interactions. Heparin, dextran sulfate (5000 mw), and chondroitin sulfate C (weakly) impaired the interaction of non‐physiological (unfractionated) properdin with sulfatide, but had no effect on physiological properdin binding,^64^ suggesting results from studies that utilized unfractionated properdin may not be applicable to the pattern of binding exerted by physiological properdin oligomers. Future studies aimed at determining potential differences between the ability of purified physiological and non‐physiological properdin, as well as serum‐ and neutrophil‐derived properdin, to bind GAG preparations could validate results from the aforementioned studies and provide key information about the binding qualities of the different properdin sources.

## 6 Properdin and thromboinflammation

Within the past decade, there has been a renewed interest in evaluating the role of properdin in disease pathogenesis. This has led to the development of properdin‐deficient mice, which have been utilized in murine models of asthma,^103^, ^104^ arthritis,^77^, ^105^, ^106^ AAA,^99^, ^103^ non‐septic^107^ and septic^74^ shock, and C3 glomerulopathy.^36^, ^49^ These studies have provided key information about the breadth of diseases exacerbated by properdin function and many have been reviewed elsewhere.^71^, ^108^, ^109^ Here, we will focus on the interactions of human properdin with platelets and neutrophils and its potential impact on thromboinflammation in the vasculature. To facilitate discussion, we begin with a brief introduction about platelet/granulocyte aggregates.

### 6.1 Platelet/granulocyte aggregates

**Figure 7:**
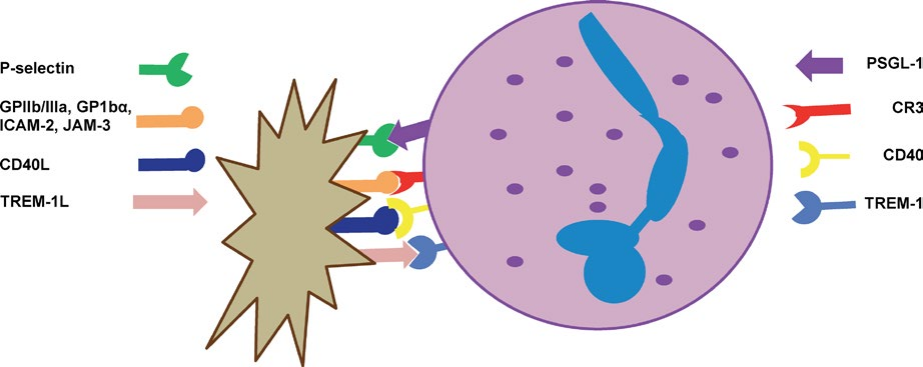
Main physical contacts governing platelet/granulocyte aggregate interactions. P‐selectin initially tethers activated platelets to granulocytes via binding to PSGL‐1. This interaction leads to an intracellular signaling cascade that activates and increases the expression of CR3 on granulocytes. CR3 binds multiple ligands on platelets including GPIIb/IIIa through a fibrinogen bridge, GP1bα directly and via a HMWK bridge, ICAM‐2, and JAM‐3. CD40L and TREM‐1L, expressed on activated platelets, enhance granulocyte functions via binding to their granulocyte counter‐receptors, CD40 and TREM‐1, respectively

Platelets not only are key mediators of vascular hemostasis as well as pathologic thrombosis, but they secrete multiple proinflammatory mediators, many of which recruit and activate neutrophils.^110^ While neutrophils are key players in the innate immune response, they also express tissue factor to aid in thrombi formation^111^, ^112^ and are capable of activating platelets via secretion of platelet activating factor^113^, ^114^ or protease‐mediated cleavage of thrombin receptors on platelets.^115^, ^116^ Platelets can directly bind and recruit neutrophils to damaged vasculature, as well as form stable aggregates in the circulation, denoted here as platelet/granulocyte aggregates (PGAs).^117^, ^118^, ^119^ Primary contacts between platelets and neutrophils involve an initial tethering event between platelet P‐selectin and P‐selectin glycoprotein ligand‐1 (PSGL‐1) on neutrophils. This stimulates an intracellular signaling cascade in neutrophils that results in the upregulation and activation of complement receptor 3 (CR3; Mac‐1; CD11b/CD18) on the neutrophil surface.^120^, ^121^, ^122^ CR3 binds to multiple receptors on platelets to form stable aggregates, including GPIIb/IIIa via a fibrinogen bridge,^123^ GP1bα alone,^124^ or with a high‐molecular‐weight kininogen (HMWK) bridge,^125^ intercellular adhesion molecule‐2 (ICAM‐2),^126^, ^127^ and junctional adhesion molecule‐3 (JAM‐3)^128^ (Fig. 7). Additionally, CD40L and TREM‐1L, expressed on activated platelets, enhance neutrophil functions via binding to their neutrophil counter receptors, CD40, and TREM‐1, respectively.^129^, ^130^ Circulating PGAs have an increased propensity to bind to the vasculature due to the adhesive properties of platelets, which can lead to thromboinflammatory effects away from the initial site of formation.^131^ Platelet binding to neutrophils (either in circulation or on the vasculature) also enhances neutrophil inflammatory responses, resulting in (i) increased secretion of proinflammatory cytokines/chemokines and damaging proteases; (ii) upregulation of adhesion molecules on neutrophils to promote infiltration into the vasculature; (iii) increased production of reactive oxygen species; and (iv) increased tissue factor expression to enhance thrombi formation.^117^, ^118^, ^119^ Therefore, excess PGA formation has significant pathologic potential, which is reflected in the beneficial effects of limiting PGA formation in various animal vascular injury models.^131^, ^132^, ^133^, ^134^ Furthermore, excess PGA formation is a phenomenon observed in patients suffering from diverse chronic inflammatory diseases, including acute coronary syndromes,^135^ inflammatory bowel disease,^136^ inflammatory lung disease,^137^ and diabetes.^138^

### 6.2 Platelets and complement

Platelet activation is a prerequisite for PGA formation, and activated platelets have extensive and dynamic interaction with the complement system. Thorough explanations of these interactions and their implications in disease pathogenesis have been reviewed elsewhere.^139^, ^140^, ^141^ Here, we consider the platelet surface as a vehicle for enhancing local complement activation to influence the interaction between platelets and neutrophils.

**Figure 8:**
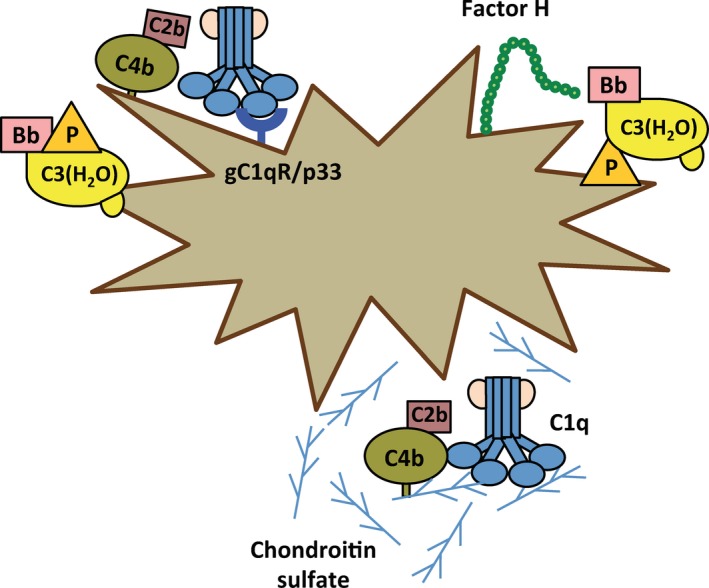
Complement activation on platelets. Platelets activate both the alternative and classical pathways on their surface. Expression of the surface receptor, gC1qR, is increased after stimulation with shear stress and binds C1q to initiate classical pathway activation. Chondroitin sulfate released from platelets upon activation also binds C1q to initiate classical pathway activity in the fluid phase. Physiological properdin (P) binds directly to activated platelets and recruits C3(H_2_O) to initiate alternative pathway activity. C3(H_2_O) also binds directly to activated platelets and leads to alternative pathway activation in the presence of properdin. Properdin‐mediated alternative pathway activation on platelets is controlled by Factor H

The surface of activated platelets acts as a platform for initiation of the classical 142 and alternative pathways.^31^, ^143^ Classical pathway activation occurs as a result of shear stress‐mediated upregulation of gC1qR/p33 on the platelet surface, which subsequently recruits C1q to initiate classical pathway activation.^142^ Chondroitin‐4‐sulfate, secreted from platelet α‐granules upon activation, interacts with many complement proteins^144^ and ultimately leads to classical pathway activation in the fluid phase.^145^ Alternative pathway activation was shown to be dependent on (i) C3b binding to P‐selectin (CD62P) on activated platelets,^143^ although Hamad et al.^146^ detected binding of only C3(H_2_O) to stimulated platelets independently from P‐selectin; (ii) the binding of physiological properdin oligomers directly to the platelet surface (without a requirement for C3) with subsequent recruitment of C3(H_2_O) and C3b to form a novel convertase.^31^ The latter study thus indicated a role for properdin‐initiated alternative pathway activity on platelets. Interestingly, chondroitin‐4‐sulfate, which also binds Factor H,^144^ enhanced properdin binding to activated platelets at low concentrations.^31^ While the precise molecular interplay between properdin, Factor H, and chondroitin‐4‐sulfate on the platelet surface was not determined, alternative pathway activation on thrombin‐activated platelets with or without properdin was greatly enhanced when Factor H cell‐surface protection was competitively inhibited by rH19‐20.^31^ This indicates a critical role for Factor H in controlling alternative pathway activation on platelets that may be relevant in an aHUS setting. AHUS‐related mutations in Factor H are associated with increased platelet activation due to dysregulated alternative pathway activity,^33^, ^147^ and thus the study by Saggu et al.^31^ gives rise to the possibility that inhibition of properdin could have beneficial effects on platelet activation in patients suffering from aHUS. Saggu et al.^31^ also showed that properdin‐initiated alternative pathway activity leads to complete activation of the terminal pathway, as measured by C9 deposited on the platelet surface. Complement activity on platelets is summarized in Fig. 8.

### 6.3 Properdin and neutrophils

While C5a generation was not directly measured in the study by Saggu et al.,^31^ complete activation of the terminal pathway on platelets implies that platelet‐mediated alternative pathway activation leads to the generation of C5a, a potent neutrophil chemoattractant and stimulator.^148^ Thus, complement activation on platelets at sites of vascular damage could aid in the recruitment and activation of neutrophils. C5a stimulation of neutrophils leads to the rapid secretion of properdin from secondary granules,^76^ which could promote further alternative pathway activation on the neutrophil surface.^75^ P‐selectin/PSGL‐1 interactions lead to the secretion of pentraxin 3 from neutrophil secondary granules.^149^ While properdin secretion was not directly measured in the study by Maugeri et al., given the colocalization of properdin and pentraxin 3 in neutrophil secondary granules,^76^, ^150^ it is possible that initial contacts between platelet P‐selectin and neutrophil PSGL‐1 also enable the secretion of properdin into the microenvironment. Properdin secreted from neutrophils has been shown to bind to the neutrophil surface in an autocrine or paracrine fashion,^75^, ^76^ although the mechanisms for properdin binding are unknown. Properdin‐enhanced alternative pathway activation on neutrophils serves as a positive feedback loop to enhance CR3 upregulation and promote oxidative bursts in neutrophils via the generation of C5a.^75^ Therefore, activated platelets not only directly bind neutrophils but may indirectly recruit and activate neutrophils via promoting complement activation on, or in close proximity to, their surface. In turn, activated neutrophils enhance their own activation and recruitment via the secretion of properdin that promotes alternative pathway activation on both neutrophils and platelets, and thus there is potential for significant complement‐dependent crosstalk in mediating the interactions between these cells.

### 6.4 Properdin and PGA formation

In line with this theory, researchers have elucidated roles for complement in enhancing PGA formation in human whole blood using extracorporeal circuits that simulate coronary artery bypass^151^, ^152^, ^153^, ^154^, ^155^ and ex vivo flow cytometry assays.^145^, ^156^, ^157^, ^158^, ^159^ Rinder et al.^152^, ^155^ demonstrated that PGA formation in an extracorporeal circuit was dependent on C5a‐mediated effects, a result that was also seen by Lappegard et al.^151^ using a heparin‐coated circuit. The latter study also established a role for the alternative pathway in enhancing C5a, by demonstrating the ability of an anti‐Factor D antibody to limit PGA formation and upregulation of CR3 on neutrophils.^151^ These findings were in agreement with independent studies showing that an anti‐Factor D^160^ or an anti‐properdin monoclonal antibody^161^ inhibited neutrophil and platelet activation (hallmarks of thromboinflammation) during simulated cardiopulmonary bypass.

Ruef et al.^158^ showed that unfractionated properdin could induce platelet/leukocyte aggregate (PLA) formation in human whole blood and significantly enhanced PLA formation in the presence of the weak platelet agonist, ADP in an ex vivo flow cytometry assay. Using a similar system, Blatt et al.^156^ recently addressed the role of physiological properdin oligomers in their ability to induce PGA, determining that they enhance PGA formation in the presence of thrombin receptor‐activating peptide (TRAP). Each properdin form (dimers, trimers, and tetramers) also sensitized whole blood to PGA formation when added to blood stimulated with a dose of TRAP that induced little to no PGA formation by itself. Properdin tetramers were the most potent oligomer with regard to enhancing PGA formation and alternative pathway activity. These data were in agreement with the high degree of ‘native properdin activity’ originally ascribed to properdin tetramers by Pangburn^65^ and also suggest that high levels of properdin (especially properdin tetramers) in the local microenvironment have the potential to exacerbate thromboinflammation in the presence of relatively weak platelet stimulation. In addition, it was determined that inhibition of endogenous properdin in the blood, using inhibitory anti‐human properdin monoclonal antibodies, reduced TRAP‐induced PGA formation by approximately 50% and to the same level as inhibiting all complement activity with compstatin, an inhibitor that prevents C3 cleavage.^156^ The overall effect of properdin was attributed to its ability to enhance the generation of C5a leading to the upregulation of CR3 on granulocytes,^156^ consistent with the findings from Hamad et al.^6^, ^145^, who originally demonstrated the role for C5a‐mediated upregulation of CR3 in promoting PGA formation in the whole blood ex vivo flow cytometry system.

Interestingly, Blatt et al. discovered that inhibition of the classical or alternative pathways, properdin function, or C5a‐C5aR1 interactions had approximately the same effects as inhibiting all complement activity. This indirectly indicated a regulatory, rather than initiating, role for properdin in PGA formation, which was dependent on its ability to enhance alternative pathway‐mediated amplification of complement activity mainly initiated by the classical pathway.^156^ These data suggest that properdin‐initiated alternative pathway activity on platelets plays little to no role in TRAP‐mediated PGA formation ex vivo. These data also highlight the interplay between the classical and alternative pathways and the effectiveness of targeting properdin as a therapeutic option, even when the classical pathway plays a key role in initiating complement activation.

Finally, Blatt et al. demonstrated that the effects of endogenous properdin on PGA formation were tightly controlled by Factor H cell‐surface protection. The addition of rH19‐20 to TRAP‐stimulated whole blood significantly increased PGA formation, and this increase was completely abrogated by inhibitory anti‐human properdin monoclonal antibodies.^156^ This suggests that in aHUS, a prothrombotic disease associated with decreased Factor H cell‐surface protection, increased PGA formation may account for part of the excessive thrombosis and inhibition of properdin function may have beneficial effects.

Hamad et al.^6^ also described a novel CR3‐C3(H_2_O) interaction between platelets and neutrophils that could mediate stable aggregate formation. Given the ability of properdin to recruit C3(H_2_O) to the platelet surface,^31^ it is possible that a dual mechanism for the ability of properdin to enhance PGA formation exists. Properdin may both increase the generation of C5a leading to CR3 upregulation, and directly recruit C3(H_2_O) to the activated platelet surface, providing an additional ligand for CR3 binding. Because inhibition of properdin limits CR3 upregulation on neutrophils (the critical receptor needed for binding to C3(H_2_O) on platelets),^156^ it was not possible to discern a potential role for a direct properdin‐mediated recruitment of C3(H_2_O) in the whole blood system.

## 7 Potential for properdin inhibitors as long‐term prophylactics for prevention of thromboinflammation

Because increased levels of PGA are associated with many different human diseases,^117^, ^118^, ^119^, ^135^, ^136^, ^137^, ^138^ targeting platelet/neutrophil interactions could be a widely applicable, beneficial, and long‐term therapeutic option to limit thromboinflammation in diverse clinical settings. Effective inhibition of PGA formation with minimal side effects, however, does not come without its challenges. Despite references made to the pathologic effects of PGA formation,^117^, ^118^, ^119^ platelet/neutrophil interactions are a normal process during vascular homeostasis, as neutrophils help limit platelet activation by degrading ADP, phagocytosing platelets, and releasing pentraxin 3 that limits platelet adhesion to fibrinogen.^162^ In turn, platelet‐derived inflammatory mediators help activate and recruit neutrophils in response to various pathogens.^163^ Platelet/neutrophil interactions in the vasculature are thus quite dynamic and completely inhibiting their binding long‐term by targeting P‐selectin or CR3 (key receptors on platelets and neutrophils, respectively, for mediating stable aggregate formation) could have detrimental side effects. P‐selectin deficient mice have an increased susceptibility to skin infections and show prolonged bleeding times,^164^ while CR3 has both key proinflammatory and protective immunomodulatory roles, and thus its dysfunction is associated with diverse clinical phenotypes ranging from increased risk for infection to an increased susceptibility to the development of systemic lupus erythrematosus.^165^ In addition, therapeutics that inhibit platelet activation (limiting all platelet/neutrophil interactions,^166^ such as clopidogrel), are associated with rare, but potentially life‐threatening bleeding phenotypes.^167^

The studies by Blatt et al. and Hamad et al., discussed in the previous section, showed that while inhibiting endogenous properdin, all complement activity, or specifically C5a‐C5aR1 interactions impairs PGA formation, the extent of inhibition approximates only 50% of the total TRAP‐induced PGA formation in their systems.^6^, ^145^, ^156^ This indicates that there is a significant complement‐independent portion of PGA formation that is likely mediated by direct contacts or soluble non‐complement mediators. By binding to PSGL‐1, P‐selectin induces the activation of CR3 to an intermediate‐affinity state capable of binding platelet receptors. Subsequent stimulation by chemokines induces a high‐affinity conformation for CR3.^168^ Based on these previous studies,^6^, ^145^, ^156^ it is likely that properdin‐mediated C5a aids in the transition of CR3 from an intermediate‐ to high‐affinity conformation, increasing the chance that neutrophils stably bind to platelets. This may help to explain why inhibition of properdin or C5a only limits, rather than completely abrogates, TRAP‐mediated PGA formation.

**Figure 9:**
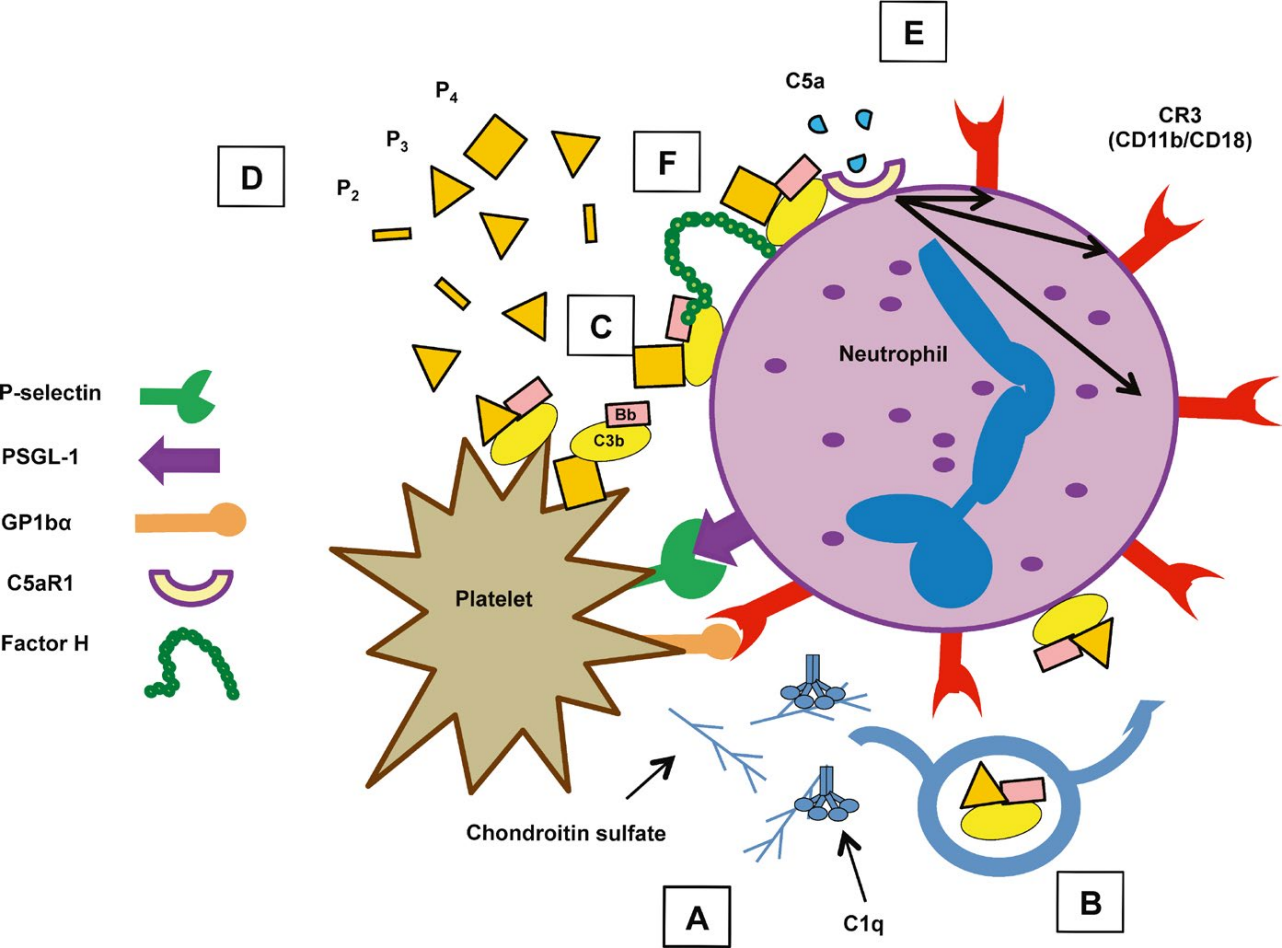
Model for the mechanism of the effects of properdin on platelet/granulocyte aggregate (PGA) formation and complement activation on each cell type. (A) Activated platelets initially tether to granulocytes via P‐selectin/PSGL‐1 interactions, where they activate the classical pathway(CP) on their surface and/or secrete chondroitin sulfate, a known activator of the CP. (B) Properdin‐enhanced alternative pathway(AP) activity amplifies CP activity initiated by platelets, leading to the deposition of C3b on the granulocyte surface. The AP can then use deposited C3b to amplify its activity directly on the granulocyte surface. The AP also activates spontaneously on (C) neutrophils and activated platelets, and (D) AP activity is enhanced by high levels of properdin oligomers (P_2_, P_3_, and especially P_4_), secreted from neutrophils. (E) Properdin‐enhanced AP activity ultimately leads to increased levels of C5a that binds to C5aR1 on neutrophils to enhance CR3 expression. (F) Factor H regulates AP/properdin‐mediated PGA formation. Figure originally printed in: Blatt et al^156^

The partial effect of complement inhibition on TRAP‐mediated PGA formation leads to the possibility that complement activity could be the force that increases granulocyte accumulation at thromboinflammatory sites to a level that becomes pathologic, rather than homeostatic. This theory is supported by epidemiologic and experimental evidence that has independently suggested roles for both granulocytes^169^ and complement^170^ in cardiovascular disease, an example of a set of diseases associated with increased circulating PGAs.^135^ Additionally, an increase in alternative pathway activity, either through the addition of exogenous properdin (especially properdin tetramers) or competitive inhibition of Factor H‐mediated cell‐surface protection, sensitizes whole blood to robust PGA formation in the presence of low doses of the platelet agonist, TRAP.^156^ The alternative pathway amplification loop can thus greatly exacerbate the response to mild thromboinflammatory stimuli when it overwhelms negative regulatory mechanisms. Theoretical reasons for this potent effect stem from the potential for multiple positive feedback loops involved in PGA formation that were alluded to in the previous section: (i) platelets and neutrophils mutually enhance each other's activation, thus any factor that enhances the activation of one cell has the potential to indirectly activate the other; (ii) platelets are both activated by complement,^171^, ^172^, ^173^, ^174^, ^175^, ^176^ and activate the alternative pathway in a properdin‐dependent manner^31^; (iii) platelet tethering to neutrophils induces the release of secondary granules^149^ containing properdin^76^ that can promote complement activation on platelets^31^ and neutrophils^75^; (iv) alternative pathway activation on neutrophils enhances neutrophil activation^75^ via mainly C5a generation; and (v) C5a, formed during alternative pathway activation on platelets or neutrophils, is a potent neutrophil chemotactic and proinflammatory agonist.^4^ Inhibition of properdin may break this potent positive feedback loop, preventing uncontrolled activation of alternative pathway activity and allowing direct platelet‐mediated responses (rather than complement‐mediated enhancement) to govern the extent of PGA formation. A model for these interactions is summarized in Fig. 9.

The consequence of inhibiting properdin in the presence of impaired Factor H cell‐surface protection was discussed above for the PGA model,^156^ and was also demonstrated in a different model by Lesher et al.^49^, who showed that inhibition of properdin in the presence of rH19‐20 prevented lysis of human and murine erythrocytes by sera. We have compared the inhibition of properdin to inhibition of C5 on sheep erythrocytes using the same rH19‐20‐mediated hemolysis assay as originally described,^24^ and found properdin inhibitors to be approximately fourfold more effective than C5 inhibitors in preventing hemolysis (N. Galwankar, H. N. Emch, S. K. Pathan, A. Z. Blatt, C. Cortes, and V. P. Ferreira, unpublished data). This has potentially significant clinical relevance, as the anti‐C5 monoclonal antibody, eculizumab, is currently the only therapeutic on the market for the treatment of aHUS. Efforts are under way in our laboratory to further investigate the role of PGA formation in aHUS and the potential benefits of properdin inhibition.

Inhibition of properdin may limit complement activity initiated by any of the three complement pathways due to the amplifying ability of the alternative pathway. Examples of this cooperative effect were found when inhibition of endogenous properdin and inhibition of the classical pathway both limited TRAP‐mediated PGA formation by approximately 50%.^156^ Given that the mechanism for the effect of properdin on TRAP‐mediated PGA formation was related to its ability to increase C5a generation, these results were in agreement with findings from the studies by Harboe et al.^14^ that showed the alternative pathway accounted for approximately 80% of terminal pathway activation when complement activity was initiated by either the classical or lectin pathway.^15^ A clear example of a beneficial effect of inhibiting properdin in an in vivo disease model in which complement activity is not initiated by the alternative pathway comes from investigating the role of complement in a murine model of AAA.^99^, ^177^ Not only did these studies show that properdin‐deficient mice were resistant to AAA development,^99^ a vascular inflammatory disease characterized by neutrophil infiltration, but it also showed that complement activity was initiated by lectin pathway‐mediated activation following the deposition of anti‐fibrinogen natural antibodies.^177^ The fact that properdin inhibition was equally effective as lectin pathway inhibition in this model gives credence to the possibility that properdin inhibitors can have potent long‐term effects in inflammatory vascular disorders, regardless of the mechanism by which complement activity is initiated. While the ex vivo data support a role for properdin‐enhanced generation of C5a, properdin inhibition would limit the generation of all complement effector molecules, and the potential in vivo effects of this should be considered. Decreased C3b and iC3b generation may limit neutrophil and monocyte infiltration into inflamed vasculature,^178^, ^179^ while decreased C3a levels would limit vasodilatory and proinflammatory effects on the vasculature.^4^

A potentially attractive therapeutic strategy may be the use of properdin inhibitors in combination with C5a inhibitors (C5 cleavage inhibitors or C5a receptor antagonists). Properdin inhibitors would limit the generation of both C5a and MAC, while C5 inhibitors or C5a receptor antagonists could account for potential C5 cleavage by thrombin or other coagulation enzymes.^180^, ^181^ To our knowledge, a therapeutic regimen such as this has never been tested in clinical trials. With the rate of advances in understanding the roles of properdin and C5a in various disease models, these two types of inhibitors could be employed simultaneously in a way that potentially lowers the effective dose of each inhibitor, thus limiting potential side effects.

To date, no properdin inhibitors have been described that can specifically target different properdin oligomers. Given the enhanced functionality of properdin tetramers compared with other oligomers,^65^, ^156^ inhibitors that specifically target properdin tetramers may be especially useful therapeutics. Development of such therapeutics would be challenging, since the properdin oligomers are identical except for the unique geometric constraints found at the vertices formed between two monomers.^54^, ^59^ If novel inhibitors could be developed, however, they would greatly facilitate the understanding of properdin biology via detailed characterization of their binding sites on properdin and their effects on properdin function and ligand binding. Salp20, a saliva protein isolated from the tick *Ixodes scapularis*, is an example of perhaps the first non‐antibody properdin inhibitor. Not only does Salp20 inhibit properdin function, it actively dissociates properdin from alternative pathway convertases.^182^ Salp20 inhibits LPS‐induced alternative pathway activity in vivo in mice and ameliorates disease pathogenesis in murine models of OVA‐induced asthma and AAA,^103^ and thus it is an excellent starting point for the future development of potent, non‐immunogenic human properdin inhibitors.

### 7.1 Potential consequences of properdin inhibition

Despite potential effects of properdin inhibition on the generation of all complement effector molecules, the only documented consequence of properdin deficiency in humans is an increased susceptibility to *Neisseria meningitidis* meningitis and septicemia. Antibodies produced following vaccination with the available vaccines have largely prevented these life‐threatening conditions.^183^ Therefore, effective strategies to mitigate the only documented consequence of properdin deficiency in humans already exist.

In vivo mouse models have provided evidence for other potential consequences of systemic, long‐term properdin inhibition. P^−/−^ mice exhibited worsened colitis, associated with decreased infiltration of neutrophils into the intestine and corresponding increases in bacterial burden in an IL‐10^−/−^ model of inflammatory bowel disease.^184^ Properdin deficiency was also shown to be detrimental in a mouse model of infectious colitis utilizing *Citrobacter rodentium*. Increased pathogenesis in this model was related to an *increase* in infiltrating neutrophils and macrophages as a result of a decreased ability of intestinal epithelial cells to control initial bacterial burden, which was shown to be dependent on properdin‐mediated generation of C5a.^185^ While these studies both showed worsened outcomes with properdin deficiency, the detrimental effect of the absence of properdin was due to very different mechanisms in these complex diseases. These studies, thus, highlight the context‐specific effects of properdin inhibition, which would not be universally applicable to all patients, in particular those that are normally properdin‐sufficient, and could potentially be alleviated by careful clinical monitoring.

Steiner et al.^186^ found a protective role for properdin in the development of early atherosclerotic lesions in male mice fed a low‐fat diet, but this protective function was eliminated upon feeding mice a high‐fat diet. While these results are intriguing, due to the strict criteria for the protective effect of properdin (only male mice fed a low‐fat diet), it is uncertain how properdin inhibition would affect the development of atherosclerosis, a complex inflammatory disease associated with multiple risk factors, in humans.

Properdin deficiency improved outcomes in a murine model of zymosan‐induced non‐septic shock, but exacerbated disease in LPS‐induced non‐septic shock.^107^ This study serves as another example of context‐specific roles for properdin that would need to be clinically monitored. It should be noted, however, that LPS is a potent neutrophil activator that induces full neutrophil degranulation.^187^ Thus, transiently increased levels of properdin due to massive LPS‐mediated neutrophil activation during non‐septic shock may compensate for decreased properdin function due to properdin inhibitors in patients that are normally properdin sufficient.

## 8 Understanding the interplay between properdin and Factor H in the microenvironment

Studies by Saggu et al.^31^ and Blatt et al.^156^ highlight the opposing roles of properdin and Factor H in the microenvironment. Aside from binding to key complement components in order to carry out their regulatory functions, both properdin and Factor H bind to a diverse set of ligands other than complement proteins. In addition to sulfatides and the polyanionic structures already mentioned, unfractionated properdin has recently been shown to bind to neutrophil myeloperoxidase,^87^ Factor H‐related protein‐5 (CFHR‐5)^81^), and monomeric C‐reactive protein (mCRP).^88^ Not only does Factor H bind mCRP,^188^ but myeloperoxidase has been shown to interfere with the interaction between mCRP and Factor H,^189^ and CFHR‐5 directly competes with Factor H for binding to pentraxin 3, thus limiting its function.^190^ Potential competition between properdin, Factor H, and these molecules only complicates the understanding of the mechanisms for regulation of the alternative pathway in the microenvironment. Future studies should aim to characterize (i) the binding properties of physiological and/or neutrophil‐derived properdin (instead of unfractionated) to acute phase reactants (e.g. mCRP and other pentraxins) and neutrophil proteases (e.g. myeloperoxidase), and (ii) the functional consequences of these interactions on alternative pathway activity, especially in the context of known disease mutations or polymorphisms associated with dysregulated alternative pathway activity, such as in aHUS or C3 glomerulopathies.^37^

Factor H also binds to many of the same polyanions associated with properdin binding, including heparin,^50^, ^191^, ^192^, ^193^, ^194^, ^195^, ^196^, ^197^, ^198^ heparan sulfate,^102^, ^193^, ^194^, ^196^ and chondroitin sulfate C.^26^, ^199^ Association with polyanions increases Factor H regulation on surfaces by directing it to specific cells^50^, ^193^ and inducing oligomerization via the C‐terminal domains.^200^ Zaferani et al. and Nagamachi et al. determined that unfractionated properdin and Factor H do not compete with each other for binding to proximal tubular epithelial cells,^102^, ^201^ but whether physiological properdin and Factor H compete for binding to these or other polyanions is unknown. In addition, it is not known whether weak properdin interactions with polyanions could impair Factor H oligomerization. Understanding the different structural characteristics that preferentially recruit properdin or Factor H could reveal novel ways to alter the level of alternative pathway activity by preferentially recruiting, or inhibiting, either properdin or Factor H to thromboinflammatory locations. For instance, Zaferani et al.^102^ were able to inhibit the binding of unfractionated properdin, but not Factor H, to HK2 cells with low sulfated anticoagulant heparinoids. While the clinical relevance of this finding is yet to be determined, it serves as a promising example for future studies aimed to identify differences in the molecular characteristics governing recruitment of these powerful alternative pathway regulators.

Interesting studies by Ruseva et al.^36^ and Lesher et al.^49^ highlighted the interplay between properdin and Factor H in controlling global complement activity in murine models of C3 glomerulopathy. Both studies analyzed the effects of properdin deficiency in mice with complete^36^ or severely impaired^49^ Factor H fluid‐phase regulation. In either scenario, properdin deficiency was severely detrimental, causing exacerbated disease pathogenesis. Properdin deficiency altered the ratio of fluid‐phase vs cell‐surface alternative pathway activation,^49^ and C5 consumption was noted in the complete absence of properdin and Factor H.^36^ Collectively, these studies represent a clear example of the complex relationships between properdin and Factor H, which must be understood, including taking into account the differences between complement regulation between mice and humans, in order to reduce the chance of profound and unexpected consequences of properdin inhibition. DDD resulting from Factor H dysfunction is an extremely rare disease,^202^ but these intriguing results by Ruseva and Lesher et al. deserve clinical consideration. Because properdin and Factor H are primarily produced by different cell types (properdin by leukocytes, Factor H by hepatocytes), it remains to be determined if preferential dysfunction of leukocytes (such as in chemotherapy) or hepatocytes (such as in cirrhosis) affects the balance between properdin and Factor H in regulating the alternative pathway and if there are associated consequences in particular disease models.

Given the important role of Factor H in tightly regulating properdin‐mediated effects on PGA formation^156^ and alternative pathway activation on platelets,^31^ even when membrane‐bound complement regulatory proteins are present, promotion of Factor H activity on cell surfaces is another plausible therapeutic option for treating or preventing thromboinflammation. Novel therapeutics like TT30^203^ and mini‐Factor H^204^, ^205^ function by linking the N‐terminal regulatory domains of Factor H to another protein that will direct the molecule to inflammatory sites where complement is activating. In TT30, Factor H N‐terminal domains are linked to CR2 to specifically target the molecule to sites of C3d accumulation.^203^ Mini‐Factor H uses the intrinsic cell‐localizing capabilities of the Factor H C‐terminus, by linking the N‐terminal regulatory domains directly to domains 19–20.^204^, ^205^ An exciting possibility is the potential use of properdin inhibitors in conjunction with these novel therapeutics. Extra control exerted over alternative pathway activation by TT30 or mini‐FH may augment the effects of properdin inhibition, although studies need to be conducted to determine potential synergistic effects. Combined use of properdin inhibitors and Factor H promoters may be particularly attractive for the treatment of patients containing aHUS‐related mutations in Factor H. Factor H promoters could directly compensate for the intrinsic defect in Factor H cell‐surface protection, while properdin inhibitors, which have proven to be effective at limiting TRAP‐mediated PGA formation^156^ and complement activation on human erythrocytes^49^ when Factor H regulation is impaired, could limit or prevent disease flare‐ups resulting from alternative pathway activation that overwhelms the level of regulation provided by Factor H promoters.

## 9 Promoting properdin activity on pathogens

Much of this review has been dedicated to the potential utility of inhibiting properdin in thromboinflammatory diseases and the associated consequences; however, properdin undoubtedly is a key weapon in the fight against invading microorgansims. A murine model of polymicrobial sepsis showed increased morbidity and mortality in the presence of genetic properdin deficiency,^74^ and properdin enhances complement activation on *Chlamydia pneumoniae*,^82^ *Neisseria meningitidis* and *gonorrhoeae*,^93^, ^206^ fungal glycans,^96^ and *Escherichia coli*.^74^, ^89^, ^97^ Therefore, finding ways to direct properdin activity to the microbial surface could provide novel ways to resolve infections.

Recombinantly produced properdin, predominantly containing non‐physiological aggregates, enabled opsonization of *Streptococcus pneumoniae* and *Neisseria meningitidis* by human serum. In the case of *Neisseria meningitidis*, the recombinant properdin also mediated in vitro killing in serum. The injection of recombinant properdin was subsequently shown to have beneficial effects in murine infection models with both pathogens.^207^ Spitzer et al.^89^ showed that a single chain antibody (scFv) targeted to murine or human erythrocytes and linked to properdin could induce complement activation on the erythrocytes. Utilizing this targeting strategy against microorganisms could prove to be effective, especially if the scFv is directed against bacterial proteins known to bind complement regulatory proteins. For instance, *Neisseria meningitidis* expresses several proteins that recruit Factor H to its surface,^208^, ^209^, ^210^ and thus scFv's directed against Factor H‐binding proteins could promote alternative pathway activity on the bacteria by simultaneously increasing properdin activity and decreasing Factor H regulation. This strategy would also likely bypass any need for antibody‐dependent classical pathway initiation due to the potent complement initiation properties of scFv‐properdin.^89^ Many bacteria other than Neisseria species including *Borrelia burgdorferi*,^211^ *Yersinia enterocolitica*,^212^ and *Streptococcus pyogenes* 213 bind Factor H (reviewed in 16) to protect themselves from the alternative pathway, therefore scFv‐properdin targeted against Factor H‐binding proteins could be an efficient strategy for treating infections resulting from a diverse array of pathogens.

## 10 Concluding remarks

Properdin is a powerful inflammatory modulator that is tightly regulated by Factor H and has the ability to both initiate and positively regulate the alternative pathway amplification loop for all complement activity. While current evidence is lacking for an initiating role for properdin in vivo or ex vivo, there remains a possibility that properdin initiation enables activation of the alternative pathway on the surface, but quickly becomes irrelevant upon deposition of multiple C3b molecules, allowing for its more prominent role as a positive regulator. With the development of novel reagents that can differentiate between initiating and regulatory roles of properdin, as well as discern properdin function resulting from different properdin oligomers or sources, critical advances will be made in understanding the complex biology of properdin in the context of diverse inflammatory and thromboinflammatory environments. A better understanding of properdin biology will open the door for the generation of novel properdin inhibitors that can be employed alone or in combination with other complement inhibitors to prevent or limit disease pathogenesis. By studying the molecular basis for the interplay between properdin and Factor H, we will better be able to predict the potential effects of either inhibiting or promoting properdin activity and could develop strategies to maximize effects on the alternative pathway by employing methods that simultaneously inhibit properdin and promote Factor H activity, or vice versa. These types of strategies may find particular usefulness in the clinic to limit inflammation in diseases marked by excessive alternative pathway activation or to treat microbial infections. With these thoughts in mind, we can collectively contribute to the knowledge required to make significant advances in the treatment or prevention of multiple disease etiologies.

## Conflict of interest

The authors declare no financial or commercial conflict of interest
